# Development of an influenza D virus with an eight- or nine-segment genome

**DOI:** 10.1038/s41598-025-34838-y

**Published:** 2026-01-08

**Authors:** Hiroho Ishida, Hironobu Murakami, Shuntaro Mizuno, Misa Katayama, Wataru Sekine, Kosuke Ohira, Akiko Takenaka-Uema, Shin Murakami, Makoto Nagai, Taisuke Horimoto

**Affiliations:** 1 School of Veterinary Medicine, Azabu University, Sagamihara, 252-5201 Kanagawa Japan; 2 Laboratory of Veterinary Microbiology, Graduate School of Agricultural and Life Sciences, The University of Tokyo, Bunkyo-ku, Tokyo, 113-8657 Japan

**Keywords:** Influenza d virus, Reverse genetics, M1, P42, M2, M1′, 8-segment, 9-segment, Vaccines, Microbiology, Virology, Influenza virus

## Abstract

Influenza D virus (IDV) contains seven genome segments. The M and NS segments are regulated via splicing to express two proteins each (P42 and M1 from the M segment and NS1 and NS2 from the NS segment). Previously, we created an eight-segment IDV by separating the *NS1* and *NS2* genes into independent monocistronic segments. In this study, we designed another eight-segment IDV with two divided M segments, transcribing either P42 or M1 mRNA independently. Specifically, we constructed two plasmids for reverse genetics: one for viral RNA (vRNA) synthesis of the P42 segment with silent mutations at the splicing donor site and one for vRNA synthesis of the M1 segment with deletion of the intron in the M segment. We successfully created the virus via reverse genetics using these two segments and six other vRNA synthesis plasmids (PB2, PB1, P3, HEF, NP, and NS). Furthermore, we generated a nine-segment IDV including the P42, M1, NS1, and NS2 segments. These systems enable independent functional and interactive analyses of M and NS segment-encoded proteins for viral infectivity. Additionally, the eight- and nine-segment IDVs exhibited significantly reduced replication in cell culture compared with wild-type IDV, suggesting a strategy for attenuated live vaccine development.

**Supplementary Information:**

The online version contains supplementary material available at 10.1038/s41598-025-34838-y.

## Introduction

Influenza D virus (IDV), a member of the Orthomyxoviridae family, was first isolated in the United States in 2011 from pigs with respiratory symptoms^1^. Subsequent epidemiological analyses identified cattle as the main hosts^2,3^, with the virus being detected in cattle from North America^2–7^, South America^8,9^, Europe^10–14^, Asia^15–19^, and Africa^20,21^. Additionally, antibodies against IDVs have been detected in feral pigs^22^, sheep^23,24^, goats^23,24^, horses^25^, dromedary camels^20,26^, deer^27^, and humans^28,29^. These findings suggest that IDVs are distributed globally in multiple hosts. IDV infection can cause mild to moderate respiratory illness in cattle, and it has been identified as a risk factor for bovine respiratory disease complex (BRDC)^5,30^, which is the most common and expensive disease affecting the cattle industry^31–33^. Considering the association between IDV and BRDC, elucidating the molecular characteristics of IDV and improving strategies for IDV vaccine development could contribute to improved BRDC control.

Influenza A and B viruses (IAV and IBV, respectively) possess eight-segment negative-sense RNA genomes (PB2, PB1, PA, HA, NP, NA, M, and NS), whereas influenza C virus (ICV) and IDV possess seven-segment genomes (PB2, PB1, P3, HEF, NP, M, and NS)^34^. However, our previous study revealed that eight ribonucleoproteins (RNPs) are actively incorporated into single ICV and IDV particles^35^. We also successfully produced IDV with eight-segment viral genomes, including two artificial NS1 and NS2 segments, which were obtained by modifying the NS segment via reverse genetics^36^. The eight-segment IDV with two divided NS segments exhibited lower replication efficiency than wild-type IDV, suggesting that IDV has an undefined tolerance packaging mechanism for viral genomes with more than eight segments, and establishing a system for generating IDVs with increased numbers of genome segments by modifying another genome segment could expand the strategies for attenuating IDV.

The NS segment-encoded mRNA splicing modes for NS1 and NS2 are equivalent for all influenza viruses^2,37–39^. However, the M segment-encoded mRNA splicing modes for M1 and M2 differ among different influenza virus types. In ICV and IDV, the M segment encodes the P42 protein, which is translated from unspliced mRNA, and the M1 protein, which is translated from spliced mRNA^2^. P42 is further processed via cleavage by signal peptidase in the endoplasmic reticulum lumen into M1′ (also called P31 protein) in the N-terminal region and M2 in the C-terminal region^40,41^. M1 is a structural protein that lines the envelope, whereas M2 is a transmembrane ion channel^42,43^. Due to the overlapping sequence regions between the *P42* and *M1* genes, it is difficult to apply reverse genetics as a mutational approach for independent functional analyses of each protein for intact IDV.

In the present study, we constructed another eight-segment IDV with divided M segments (P42 and M1) to enable independent functional analysis of the M1 and P42 proteins. Furthermore, we attempted to create a nine-segment IDV featuring divided M (P42 and M1) and NS (NS1 and NS2) segments. We discussed the utility of these artificial multisegmented IDVs as tools for molecular analyses of IDV and as potential candidates for live attenuated vaccine development.

## Results

### Creation of an eight-segment IDV with divided M segments

**Fig. 1:**
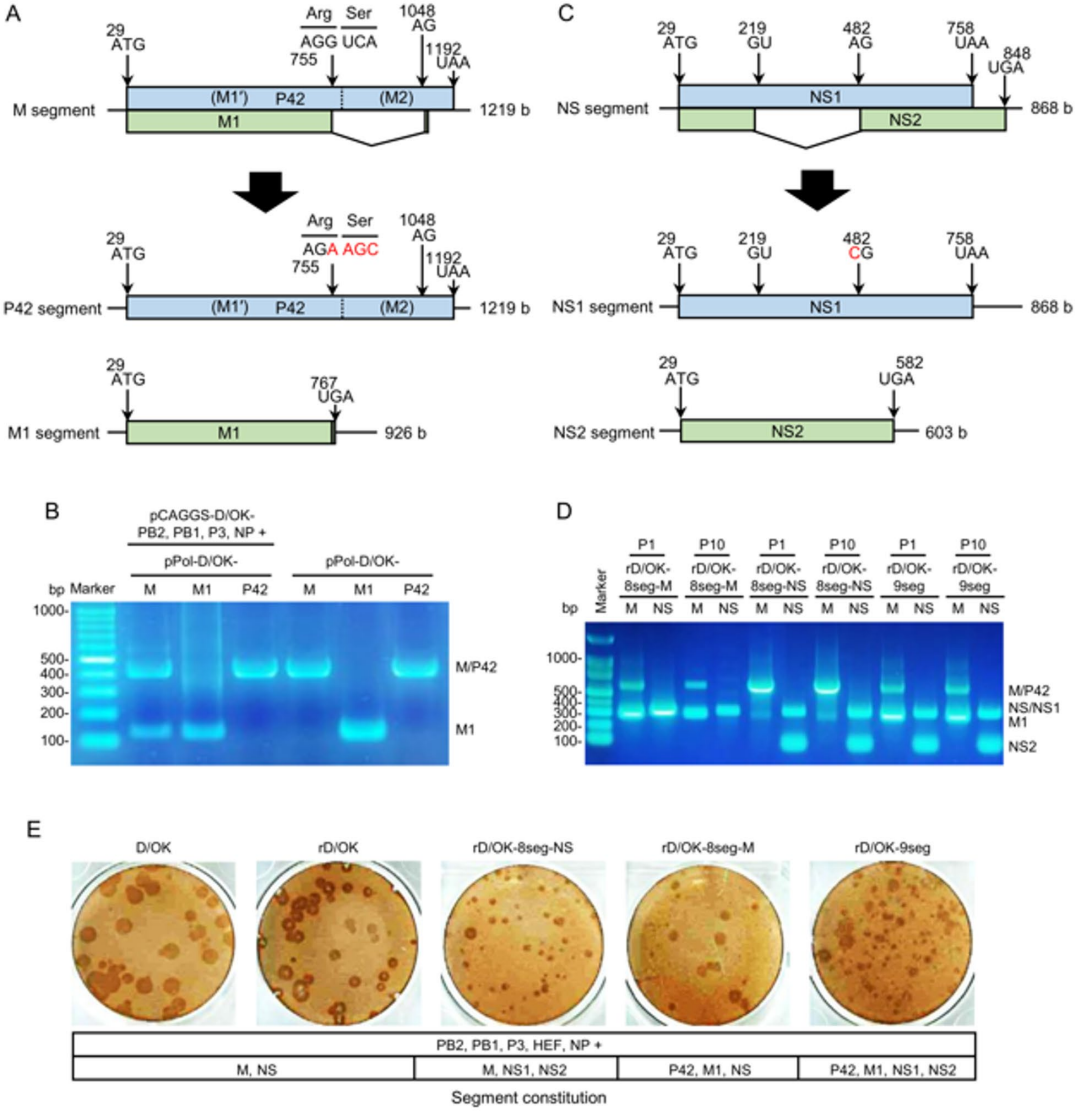
Generation of eight- or nine-segment IDVs. (**A**) We created pPol vRNA synthesis plasmids for transcribing the monocistronic P42 or M1 segment by engineering the M segment of D/OK. P42- (putative M1′ and M2) and M1-specific regions are presented in blue and green, respectively. Each number indicates the nucleotide position, and the red letters indicate the substituted nucleotides. (**B**) pPol plasmids for the M, M1, or P42 segment were transfected with four protein expression plasmids (PB2, PB1, P3, and NP) into HEK293T cells. RNA was extracted from the cells, reverse-transcribed using an oligo(dT) primer, and the cDNAs were amplified using PCR with *M* gene-specific primers (forward: 5′-AGCAA TGGAA GGGCA CCTGC-3′; reverse: 5′-TCTCG CCTGG ATTCC AGGAC-3′), followed by electrophoresis on a 1% agarose gel. The expected product sizes were 429 bp (M and P42) and 136 bp (M1). The last three lanes present positive controls, in which each pPol plasmid (M, M1, and P42) was directly used as a PCR template without reverse transcription. The uncropped gel image is provided in Supplementary Fig. S1A. (**C**) The monocistronic NS1 or NS2 segment previously created by our group by engineering the NS segment of D/OK. NS1- and NS2-specific regions are presented in blue and green, respectively. The red letters indicate the substituted nucleotides. (**D**) Eight-to-nine-segment vRNA synthesis plasmids [PB2, PB1, P3, HEF, NP, M (or M1 and P42), NS (or NS1 and NS2)] were co-transfected into HEK293T cells together with four protein expression plasmids. The supernatant was then inoculated into ST cells and serially passaged 10 times. The vRNA encoding M1, P42, NS1, or NS2 was detected in the supernatant of ST cells at passage 1 (P1) and passage 10 (P10) using RT-PCR with *M* gene primers (forward: 5′-TCACA AGATG TCAAT GTCTG-3′; reverse: 5′-ATTCC AGGAC CATTA TATCC-3′) and *NS* gene primers (forward: 5′-CGAGA ACTTG GGAAG ATGC-3′; reverse: 5′-TTCTT AAGGC AGTAA GCTGG-3′), followed by electrophoresis on a 1% agarose gel. The expected product sizes were 590 bp (M and P42), 297 bp (M1), 328 bp (NS and NS1), and 63 bp (NS2). The uncropped gel image is provided in Supplementary Fig. S1B. (**E**) Representative plaque morphologies of wild-type D/OK, D/OK rescued by reverse genetics (rD/OK), rD/OK-8seg-NS, rD/OK-8seg-M, and rD/OK-9seg in ST cells was determined by immunostaining with anti-D/OK mouse polyclonal antibody. The segment constitution of each virus is presented at the bottom.

To create a recombinant IDV with two divided M segments through reverse genetics^44^, we modified the original M segment of D/swine/Oklahoma/1334/2011 (D/OK) into two segments: one transcribing the *P42* gene alone (P42 segment) and the other transcribing the *M1* gene alone (M1 segment). Thus, we constructed a viral RNA (vRNA) synthesis plasmid for the P42 segment by inactivating the splicing donor site of M1 pre-mRNA and a vRNA synthesis plasmid for the M1 segment by deleting the intron sequence of M1 pre-mRNA (Fig. 1A). To verify the splicing defect, these two plasmids were transfected into human embryonic kidney 293 T (HEK293T) cells with or without expression plasmids for each of the viral polymerase complex components (PB2, PB1, and P3) and the capsid protein (NP), and their transcripts were then analyzed using RT-PCR (Fig. 1B). As expected, the P42 segment caused a defect in *M1* gene splicing. Thereafter, we transfected HEK293T cells with these two modified plasmids together with PB2, PB1, P3, HEF, NP, and NS vRNA synthesis plasmids or PB2, PB1, P3, and NP protein expression plasmids. Subsequently, we exposed swine testicle (ST) cells to the supernatant of the transfected cells to assess cytopathic effects (CPEs). We detected M1 and P42 vRNAs in the rescued virus in the cell supernatant (Fig. 1D), which formed plaques (Fig. 1E), but this was not observed when either the M1 or P42 segment synthesis plasmid was transfected together with the other six vRNA synthesis and four protein expression plasmids. These findings suggested the successful generation of eight-segment IDV with divided M segments (named rD/OK-8seg-M). After 10 passages, rD/OK-8seg-M retained divided M segments (Fig. 1D).

### Creation of a nine-segment IDV with divided M and NS segments

We previously developed an eight-segment IDV with divided NS (NS1 and NS2) segments (named rD/OK-8seg-NS; Fig. 1C)^36^. We next created a recombinant IDV with nine segments (PB2, PB1, P3, HEF, NP, M1, P42, NS1, and NS2) via reverse genetics using nine vRNA synthesis plasmids under the same rescue conditions. We observed CPEs on ST cells and confirmed the presence of the M1, P42, NS1, and NS2 segments of the rescued virus in the cell supernatant (Fig. 1D), which formed plaques (Fig. 1E). These findings suggested the successful generation of the nine-segment IDV with divided M and NS segments (named rD/OK-9seg). After 10 passages, rD/OK-9seg retained divided M and NS segments (Fig. 1D).

### Indirect evaluation of genome-segment composition in rD/OK-8seg-M and rD/OK-9seg viruses

**Fig. 2:**
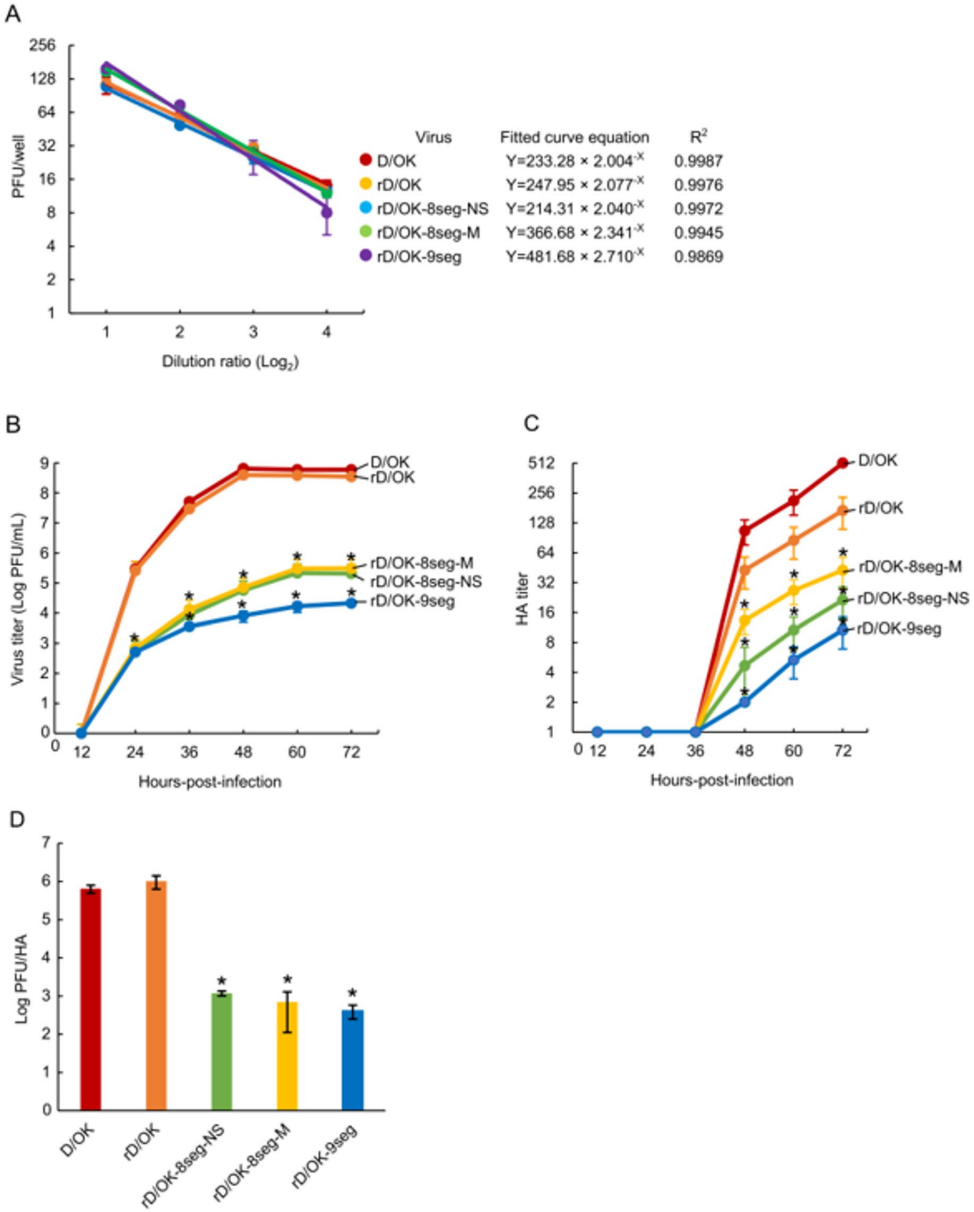
Growth properties of eight- or nine-segment IDVs. (**A**) We evaluated the correlation between the dilution ratio and the number of plaques for D/OK, rD/OK, rD/OK-8seg-NS, rD/OK-8seg-M, and D/OK-9seg. Plaque numbers at each dilution were expressed as the mean titers with standard deviations (*n* = 3). Coefficient of determination (R^2^) was determined by regression analysis for each virus, and it is presented at the right of the figure. (**B**) Growth kinetics of rD/OK-8seg-NS, rD/OK-8seg-M, and D/OK-9seg were compared with that of wild-type D/OK and rD/OK in ST cells (multiplicity of infection = 0.0001). Viral titers were determined at 12-h intervals post-infection by the plaque or (**C**) HA assay and reported as the mean titers with standard deviations (*n* = 3). The asterisk denotes significance (*P* < 0.05) compared with rD/OK as determined repeated-measures ANOVA with a post-hoc Holm’s test at each time point. (**D**) PFU/HA ratios were calculated in each virus stock (D/OK, rD/OK, rD/OK-8seg-NS, rD/OK-8seg-M, and rD/OK-9seg) in ST cells at 48 h post-infection (multiplicity of infection = 0.0001) and reported as the mean titers with standard deviations (*n* = 3). The asterisk indicates a significant difference (*P* < 0.05 by Dunnett’s test) between rD/OK and eight- or nine-segment IDVs.

To indirectly assess whether rD/OK-8seg-M and rD/OK-9seg viruses generally package complete sets of eight or nine vRNA segments, respectively, we serially diluted the virus stocks and determined infectious titers using a plaque assay (Fig. 2A). The number of plaques formed by rD/OK-8seg-M decreased proportionally with dilution, similar to wild-type D/OK. This observation supports the interpretation that rD/OK-8seg-M packages all eight RNA segments, including the M1 and P42 segments, within a single particle, because infectivity resulting from coinfection with two or more noninfectious viruses possessing incomplete genome sets, if present, would not decrease proportionally with dilution. Similarly, although the base of the formula for the approximate curve of rD/OK-9seg was 2.710 and the number of plaques decreased slightly more than the dilution rate (Fig. 2A), the results were nearly equivalent to those for wild-type D/OK and rD/OK-8seg-M. Furthermore, we generated virus-like particles (VLPs) lacking one essential segment (Table 1) and tested three coinfection models (VLP-7seg-ΔNS1 with VLP-7seg-ΔNS2, VLP-7seg-ΔP42 with VLP-7seg-ΔM1, and VLP-8seg-ΔNS1 with VLP-8seg-ΔNS2). When culture supernatants containing each VLP were coinfected into ST cells without dilution, only 0–2 plaques per well were observed, suggesting that productive coinfection occurred only at an extremely low frequency. These results suggest that most rD/OK-8seg-M and rD/OK-9seg particles may contain eight or nine segments.

**Table 1:** Genome segments incorporated into each VLP construct.

VLPs	Genome segments
VLP-7seg-ΔNS1	PB2, PB1, P3, HEF, NP, M, NS2
VLP-7seg-ΔNS2	PB2, PB1, P3, HEF, NP, M, NS1
VLP-7seg-ΔP42	PB2, PB1, P3, HEF, NP, M1, NS
VLP-7seg-ΔM1	PB2, PB1, P3, HEF, NP, P42, NS
VLP-8seg-ΔNS1	PB2, PB1, P3, HEF, NP, P42, M1, NS2
VLP-8seg-ΔNS2	PB2, PB1, P3, HEF, NP, P42, M1, NS1

### Replication properties of rD/OK-8seg-M and rD/OK-9seg

We assessed the growth kinetics of rD/OK-8seg-M and rD/OK-9seg in ST cells. The peak titer of rD/OK-8seg-M, which was equivalent to that of rD/OK-8seg-NS, was approximately 1000-fold lower than those of wild-type D/OK and rD/OK (Fig. 2B), as supported by its smaller plaque size (Fig. 1E). The peak titer of rD/OK-9seg was further reduced by approximately 10-fold compared with those of rD/OK-8seg-M and rD/OK-8seg-NS. To determine the cause of this discrepancy, we compared the ratios of the peak infectivity titer (PFU) to the HA titer of eight- and nine-segment rD/OK to wild-type D/OK (Fig. 2C), observing significantly lower ratios for the recombinant viruses (Fig. 2D). These results suggested that stocks of the recombinant more segmented viruses contained higher proportions of noninfectious particles than the wild-type viruses.

## Discussion

In this study, we used reverse genetics to create an infectious eight-segment IDV that contained artificially created monocistronic M1 and P42 segments from the original M segment and evaluated its growth characteristics. The eight-segment rescue system represents an alternative approach for evaluating the infectivity of IDV at the molecular level, as it enables independent mutational analyses of M1 and P42 (M1′ and M2) without alteration of the overlapping sequence regions. Furthermore, we successfully established a nine-segment rescue system of IDV with divided M and NS segments as an advanced reverse genetics strategy, indicating possible tolerance of IDV genome packaging in the virion and providing another tool for the molecular analysis of IDV. Although the eight- and nine-segment IDVs exhibited markedly reduced growth compared with the wild-type IDV, which may limit their utility for viral protein functional analyses, our previous study demonstrated that the eight-segment virus with divided NS segments enabled functional analysis of the N-terminal overlapping regions of the NS1 and NS2 proteins^36^. This suggests that newly generated viruses with divided M segments could also be applicable to functional studies of the respective proteins. Future research should aim to individually analyze the functions of the M1 and M2 proteins of IDV using these viruses.

Although random and selective models for genome packaging mechanisms have been proposed for IAV^45^, the latter model is favored on the basis of studies revealing the presence of segment-specific packaging signals at the 5′ and 3′ ends of each RNA segment^46–52^ and the incorporation of eight different types of RNA segments into a single virus particle^53–56^. In contrast to that of IAV, the genome packaging mechanism of IDV, a newly classified influenza virus^57^, remains largely unknown. If IDV adopts a selective genome packaging mechanism, then it will require seven different RNA segments for infectivity. However, electron microscopy revealed that IDV incorporates eight RNPs in a unique “1 + 7” pattern, similar to that of IAVs, in which seven RNPs surround the central RNP^35^. However, the specific RNA content in the particles is unknown. Therefore, IDV particles could possess the spatial capacity to accommodate eight RNA segments in a single particle. This postulation is supported by the fact that a ribosomal RNA lacking a specific packaging signal was incorporated as the eighth segment into artificial seven-segment IAV particles^55^. In fact, we generated recombinant IDVs with eight RNA segments in a single particle, including NS1 and NS2 segments in the previous study^36^ and M1 and P42 segments in the present study. Although the packaging signal sequences of the IDV M segment have not been determined, they can exist at both common ends of the M1 and P42 segments, suggesting that two different segments with the same packaging signal can be incorporated into a single particle. We hypothesized that one segment was incorporated selectively, and the other was incorporated randomly into a particle. This hypothesis is supported by our finding that rD/OK-8seg, wherein all eight segments must be incorporated into a particle for infectivity, produced more noninfectious particles than wild-type rD/OK, wherein any segment can be accepted as the eighth segment, by comparative analysis with ratios of infectivity to HA titers. The reduced growth rates of rD/OK-8seg, compared with that of the wild-type virus, might be attributable to inefficient genome packaging.

We successfully produced a nine-segment IDV that included the artificial M1, P42, NS1, and NS2 segments, indicating flexible genome packaging capacity in the virus particle, although the efficacy of incorporation of the ninth segment into the particle must be low, as indicated by the lower growth rate of rD/OK-9seg than rD/OK-8seg. Taken together, we propose an IDV genome packaging mechanism in which seven different segments are selectively incorporated into a virus particle via packaging signals, but the particle has the capacity for the random incorporation of two additional segments to acquire infectivity. Further research is needed to elucidate the genome packaging mechanism of IDV, including identification of the packaging signal sequences in each segment and observation of genomic segments within rD/OK-8seg and rD/OK-9seg using electron microscopy.

In ICV and IDV, the *M1* and *P42* genes, both encoded in M segments, have long overlapping sequences upstream of the splicing donor site for M1 pre-mRNA. After translation, the P42 protein of ICV crosses the endoplasmic reticulum membrane via two transmembrane regions, followed by cleavage by signal peptidase at the site downstream the first transmembrane region into the M1′ region and M2 protein (Supplementary Fig. S2A)^40,41^. The exact transmembrane region or cleavage site in the P42 protein of IDV has not been identified. We predicted from the ICV experiment that IDV P42 possesses two transmembrane regions rich in hydrophobic amino acids, which cross the endoplasmic reticulum membrane, and cleavage might occur between amino acids 262 and 263 with a possible motif (V-X-A) by signal peptidase^58^, generating M1′ and M2 (Supplementary Fig. S2B). Similarly, as noted for ICV, the M1′ region of IDV might be required for P42 to be located at the endoplasmic reticulum, resulting in M2 protein expression, but its function as a nonstructural protein is not clear. These predictions remain speculative and require experimental validation in future studies. Importantly, our eight-segment IDV rescue system with a divided M segment could be used to analyze the roles of M1, M1′, or M2 protein in viral infectivity independently because the intended mutations can be introduced into each protein independently, regardless of the overlapped sequence between M1 and P42.

Combined vaccines containing multiple bovine respiratory viruses and bacteria have been used to control BRDC; however, their effectiveness is limited^23–25^. One reason for this limitation might be that BRDC has been caused by agents not targeted by the current vaccines. Moreover, metagenomic analyses revealed a positive correlation between BRDC and IDV infection^5,22^. Therefore, the development of an IDV vaccine and its combined use with the current vaccine could provide more effective BRDC control. We previously established a candidate attenuated live vaccine strain of IDV with a high temperature sensitivity phenotype by introducing mutations in the *PB2* and *PB1* genes and demonstrated its efficacy against IDV infection^59^. Our eight- or nine-segment IDVs are promising as alternative attenuated live vaccine strains because of their low possibility for pathogenic revision through reassortment with circulating strains. Moreover, since these viruses retained the divided M or NS segments after 10 passages, the segment-division strategy itself appears to be stably maintained. Additional studies in relevant animal models will be required to evaluate their safety and protective efficacy as vaccine candidates.

In conclusion, we created an eight-segment IDV with divided M segments and further produced a nine-segment IDV with both divided M and NS segments. Evaluation of the virological characteristics of these more segmented IDVs has provided insights into the mechanism of genome packaging. In addition, our established eight- or nine-segment rescue system will provide a powerful tool for analyzing the individual functions of M1, P42, M1′, and M2 and the interactions among viral proteins, including NS1 and NS2. In addition, the eight- or nine-segment rescue system could lead to a new conceptual strategy for the development of live vaccines for IDV that do not affect viral antigenicity.

## Materials and methods

### Cells and viruses

HEK293T cells (RCB2202, Riken Bio Resource Research Center, Kyoto, Japan) and ST cells (CRL-1746, American Type Culture Collection, Manassas, VA, USA) were maintained in Dulbecco’s modified Eagle’s medium (Fujifilm Wako Pure Chemical, Osaka, Japan) supplemented with 10% fetal bovine serum at 37 °C. The IDV D/OK strain (GenBank accession numbers JQ922305–JQ922311) was kindly provided by Dr. Feng Li (University of Kentucky, Lexington, KY, USA). We also used the recombinant eight-segment IDV with a divided NS segment containing NS1 and NS2 (rD/OK-8seg-NS), which was generated in our previous study^36^. D/OK and rD/OK-8seg-NS were propagated in ST cells cultured in Eagle’s minimum essential medium (MEM; Fujifilm Wako Pure Chemical, Osaka, Japan) containing 0.3% bovine serum albumin (MEM/BSA) and 0.5 µg/ml l−1-tosylamido-2-phenyl chloromethyl ketone (TPCK)-trypsin (Worthington, Lakewood, NJ, USA) and stored at − 80 °C.

### Plasmid construction

vRNA synthesis plasmids (pPol-D/OK-PB2, pPol-D/OK-PB1, pPol-D/OK-P3, pPol-D/OK-HEF, pPol-D/OK-NP, pPol-D/OK-M, and pPol-D/OK-NS) that contained the cDNAs of the D/OK viral genes between the human RNA polymerase I promoter and mouse RNA polymerase I terminator and eukaryotic protein expression plasmids (pCAGGS-D/OK-PB2, pCAGGS-D/OK-PB1, pCAGGS-D/OK-P3, and pCAGGS-D/OK-NP) under the control of the chicken β-actin promoter were used for reverse genetics, as described previously^44,60^. A modified pPol-D/OK-M plasmid specifically transcribing P42 vRNA (pPol-D/OK-P42) was constructed by introducing a synonymous mutation (GTCA to AAGC) at the splicing donor site (nucleotide positions 757–760). A modified pPol-D/OK-M plasmid specifically transcribing M1 vRNA (pPol-D/OK-M1) was constructed by deleting the intron sequence (nucleotide positions 757–1049) of the M segment.

### RT-PCR

RNA was extracted from cells or viruses using TRIzol Reagent or TRIzol LS Reagent (Invitrogen, Thermo Fisher Scientific, Waltham, MA, USA), treated with recombinant DNase I (Takara Bio, Shiga, Japan), and purified using ethanol precipitation. The purified RNA was reverse-transcribed using PrimeScript Reverse Transcriptase (Takara Bio, Shiga, Japan) with oligo (dT) primers or random primers. The cDNA was amplified using PCR with GoTaq Green Master Mix (Promega, Madison, WI, USA). PCR was performed with an initial denaturation at 95 °C for 3 min; 35 cycles of 95 °C for 30 s, 51–61 °C for 30 s, and 72 °C for 1 min; and a final extension at 72 °C for 5 min. The size of the amplified products was assessed by electrophoresis using a 1.0% agarose gel. The uncropped gel images are provided in Supplementary Fig. S1.

### Reverse genetics

Reverse genetics was used to generate an eight-segment virus with a divided M segment as previously described^44^ with some modifications. Briefly, 60% confluent HEK293T cells on a six-well plate were transfected with different amounts of 12 plasmids (0.6 µg each of pPol-D/OK-PB2, pPol-D/OK-P3, and pPol-D/OK-NP; 0.1 µg each of pPol-D/OK-PB1, pPol-D/OK-HEF, pPol-D/OK-P42, pPol-D/OK-M1, and pPol-D/OK-NS; and 1.0 µg each of pCAGGS-D/OK-PB2, pCAGGS-D/OK-PB1, pCAGGS-D/OK-P3, and pCAGGS-D/OK-NP) using TransIT-293T (Mirus Bio, Madison, WI, USA) according to the manufacturer’s instructions. DNAs and 12.8 µL of the transfection reagent were mixed and incubated at 21 °C for 20 min. The mixtures were then added to the cells, which were incubated at 37 °C. The day after transfection, the supernatants were removed, and the cells were washed twice with Opti-MEM (Thermo Fisher Scientific). The cells were then incubated with 2 mL of Opti-MEM containing 0.3% BSA for an additional 2–3 days at 37 °C. The culture supernatant was collected and mixed with TPCK-trypsin (0.5 µg/mL), and the mixture was inoculated onto ST cells for 1 h. The cells were washed twice with MEM and then incubated with 2 mL of MEM/BSA containing TPCK-trypsin (0.5 µg/mL) at 37 °C. At 5 days post-infection, the supernatant was collected, and the virus (rD/OK-8seg-M) was titrated in ST cells by the plaque assay. In addition, reverse genetics was performed to generate a nine-segment virus with divided M and NS segments (rD/OK-9seg) under the same rescue conditions using 0.1 µg each of pPol-NS1 and pPol-NS2, which were used to generate rD/OK-8seg-NS in our previous study^36^, in addition to pPol-D/OK-M1 and pPol-D/OK-P42.

### Plaque assay

The plaque assay was performed as described previously^44^. Confluent ST cells on a 12-well plate were inoculated with 100 µL each of 10-fold serially diluted viruses in MEM/BSA and incubated for 1 h at 37 °C. The cells were then washed with MEM/BSA, covered with 1 mL of MEM/BSA containing TPCK-trypsin (0.5 µg/mL) and 1% Seakem GTG agarose (Lonza Japan, Chiba, Japan), and incubated at 37 °C for 3 days. Subsequently, 4% paraformaldehyde in PBS (500 µL) was added to each well to fix the cells at 4 °C overnight. After removing the paraformaldehyde and agarose, the cells were washed with PBS and permeabilized with 0.1% Triton X-100 in PBS for 15 min at 21 °C. After blocking with Block-Ace (KAC, Hyogo, Japan), the cells were incubated with anti-IDV D/OK mouse immune serum for 60 min, biotinylated anti-mouse IgG antibody (#B7264, Sigma-Aldrich, St. Louis, MO, USA) for 30 min, and a complex with streptavidin (8 µg/mL; Fujifilm Wako Pure Chemicals) and biotinylated peroxidase (4 µg/mL; Thermo Fisher Scientific) for 30 min. The plaques were visualized using a DAB peroxidase substrate kit (Vector Laboratories, Burlingame, CA, USA) according to the manufacturer’s instructions.

### VLPs

To generate VLPs lacking one of the *P42*, *M1*, *NS1*, or *NS2* genes, we used a modified reverse genetics system. HEK293T cells were transfected with the combinations of pPol plasmids listed in Table 1, together with six pCAGGS plasmids for PB2, PB1, P3, NP, M, and NS. The day after transfection, the supernatants were removed, and the cells were washed twice with Opti-MEM (Thermo Fisher Scientific). Then, the cells were incubated with 2 mL Opti-MEM containing 0.3% BSA for an additional 2–3 days at 37 °C. The culture supernatant was collected and mixed with TPCK-trypsin (0.5 µg/mL). ST cells were subsequently coinfected with three combinations (VLP-7seg-ΔNS1 with VLP-7seg-ΔNS2, VLP-7seg-ΔP42 with VLP-7seg-ΔM1, and VLP-8seg-ΔNS1 with VLP-8seg-ΔNS2), after which a plaque assay was performed.

### Hemagglutination assay

A hemagglutination (HA) assay was performed in U-bottom 96-well microplates as previously described^61^. Briefly, 2-fold serial dilutions of the supernatants in 50 µL PBS were mixed with 50 µL 0.7% chicken red blood cells and incubated for 30 min at 21 °C. HA titers were determined as the reciprocal of the highest virus dilution exhibited complete HA.

### Statistical analysis

The statistical significance of the differences in growth curves between viruses was analyzed using repeated-measures analysis of variance (ANOVA) with a post-hoc Holm’s multiple comparison test, and the significance of differences in PFU/HA ratios was analyzed using Dunnett’s test.

## Supplementary Information

Below is the link to the electronic supplementary material.

Supplementary Material 1

## Data Availability

The data used to support the findings of this study are available on request from the corresponding author, H.I.

## References

[1] Hause, B. M. et al. Isolation of a novel swine influenza virus from Oklahoma in 2011 which is distantly related to human influenza C viruses. *PLoS Pathog*. **9**, e1003176 (2013). 23408893 10.1371/journal.ppat.1003176 PMC3567177

[2] Hause, B. M. et al. Characterization of a novel influenza virus in cattle and Swine: proposal for a new genus in the Orthomyxoviridae family. *MBio* 5, e00031-14 (2014). 10.1128/mBio.00031-14 PMC3958797 24595369

[3] Ferguson, L. et al. Influenza D virus infection in Mississippi beef cattle. *Virology* **486**, 28–34 (2015). 26386554 10.1016/j.virol.2015.08.030 PMC4710178

[4] Collin, E. A. et al. Cocirculation of two distinct genetic and antigenic lineages of proposed influenza D virus in cattle. *J. Virol.* **89**, 1036–1042 (2015). 25355894 10.1128/JVI.02718-14 PMC4300623

[5] Mitra, N., Cernicchiaro, N., Torres, S., Li, F. & Hause, B. M. Metagenomic characterization of the Virome associated with bovine respiratory disease in feedlot cattle identified novel viruses and suggests an etiologic role for influenza D virus. *J. Gen. Virol.* **97**, 1771–1784 (2016). 27154756 10.1099/jgv.0.000492 PMC5772826

[6] Zhang, M. et al. The pulmonary virome, bacteriological and histopathological findings in bovine respiratory disease from Western Canada. *Transbound. Emerg. Dis.* **67**, 924–934 (2020). 31715071 10.1111/tbed.13419 PMC7168541

[7] Huang, C. et al. Emergence of new phylogenetic lineage of influenza D virus with broad antigenicity in California, united States. *Emerg. Microbes Infect.* **10**, 739–742 (2021). 33771071 10.1080/22221751.2021.1910078 PMC8043534

[8] Alvarez, I. J. et al. Seroprevalence of influenza D virus in bulls in Argentina. *J. Vet. Diagn. Invest.* **32**, 585–588 (2020). 32552516 10.1177/1040638720934056 PMC7438650

[9] Da Silva, M. S. et al. Cattle influenza D virus in Brazil is divergent from established lineages. *Arch. Virol.* **167**, 1181–1184 (2022). 35301569 10.1007/s00705-022-05416-8 PMC8929453

[10] Ducatez, M. F., Pelletier, C. & Meyer, G. Influenza D virus in cattle, France, 2011–2014. *Emerg. Infect. Dis.* **21**, 368–371 (2015). 25628038 10.3201/eid2102.141449 PMC4313661

[11] Chiapponi, C. et al. Detection of influenza D virus among swine and cattle, Italy. *Emerg. Infect. Dis.* **22**, 352–354 (2016). 26812282 10.3201/eid2202.151439 PMC4734544

[12] Flynn, O. et al. Influenza D virus in cattle, Ireland. *Emerg. Infect. Dis.* **24**, 389–391 (2018). 29350168 10.3201/eid2402.170759 PMC5782902

[13] Snoeck, C. J. et al. Influenza D virus circulation in cattle and swine, Luxembourg, 2012–2016. *Emerg. Infect. Dis.* **24**, 1388–1389 (2018). 29912692 10.3201/eid2407.171937 PMC6038750

[14] Goecke, N. B., Liang, Y., Otten, N. D., Hjulsager, C. K. & Larsen, L. E. Characterization of influenza D virus in Danish calves. *Viruses* **14**, 423 (2022). 35216016 10.3390/v14020423 PMC8880214

[15] Jiang, W. M. et al. Identification of a potential novel type of influenza virus in bovine in China. *Virus Genes*. **49**, 493–496 (2014). 25142163 10.1007/s11262-014-1107-3

[16] Murakami, S. et al. Influenza D virus infection in herd of cattle, Japan. *Emerg. Infect. Dis.* **22**, 1517–1519 (2016). 27434213 10.3201/eid2208.160362 PMC4982187

[17] Horimoto, T. et al. Nationwide distribution of bovine influenza D virus infection in Japan. *PLoS One*. **11**, e0163828 (2016). 27682422 10.1371/journal.pone.0163828 PMC5040247

[18] Zhai, S. L. et al. Influenza D virus in animal species in Guangdong province, Southern China. *Emerg. Infect. Dis.* **23**, 1392–1396 (2017). 28726609 10.3201/eid2308.170059 PMC5547803

[19] Lim, E. H. et al. First detection of influenza D virus infection in cattle and pigs in the Republic of Korea. *Microorganisms* **11**, 1751 (2023). 37512923 10.3390/microorganisms11071751 PMC10386134

[20] Salem, E. et al. Serologic evidence for influenza C and D virus among ruminants and camelids, Africa, 1991–2015. *Emerg. Infect. Dis.* **23**, 1556–1559 (2017). 28820371 10.3201/eid2309.170342 PMC5572875

[21] Molini, U. et al. First influenza D virus full-genome sequence retrieved from livestock in Namibia, Africa. *Acta Trop.* **232**, 106482 (2022). 35537488 10.1016/j.actatropica.2022.106482

[22] Ferguson, L. et al. Influenza D virus infection in feral swine populations, united States. *Emerg. Infect. Dis.* **24**, 1020–1028 (2018). 29774857 10.3201/eid2406.172102 PMC6004836

[23] Quast, M. et al. Serological evidence for the presence of influenza D virus in small ruminants. *Vet. Microbiol.* **180**, 281–285 (2015). 26414999 10.1016/j.vetmic.2015.09.005 PMC4618254

[24] Oliva, J. et al. Serological evidence of influenza D virus circulation among cattle and small ruminants in France. *Viruses* **11**, 516 (2019). 31195597 10.3390/v11060516 PMC6630579

[25] Nedland, H. et al. Serological evidence for the co-circulation of two lineages of influenza D viruses in equine populations of the Midwest united States. *Zoonoses Public. Health*. **65**, e148–e154 (2018). 29139222 10.1111/zph.12423 PMC5766371

[26] Murakami, S. et al. Influenza D virus infection in dromedary camels, Ethiopia. *Emerg. Infect. Dis.* **25**, 1224–1226 (2019). 31107233 10.3201/eid2506.181158 PMC6537730

[27] Guan, M. et al. Exposure of white-tailed deer in North America to influenza D virus. *Virology* **573**, 111–117 (2022). 35751973 10.1016/j.virol.2022.06.007

[28] White, S. K., Ma, W., McDaniel, C. J., Gray, G. C. & Lednicky, J. A. Serologic evidence of exposure to influenza D virus among persons with occupational contact with cattle. *J. Clin. Virol.* **81**, 31–33 (2016). 27294672 10.1016/j.jcv.2016.05.017

[29] Trombetta, C. M. et al. Influenza D virus: serological evidence in the Italian population from 2005 to 2017. *Viruses* **12**, 30 (2019). 31892120 10.3390/v12010030 PMC7019439

[30] Ng, T. F. et al. A metagenomics and case-control study to identify viruses associated with bovine respiratory disease. *J. Virol.* **89**, 5340–5349 (2015). 25740998 10.1128/JVI.00064-15 PMC4442534

[31] Hilton, W. M. BRD in 2014: where have we been, where are we now, and where do we want to go? *Anim. Heal Res. Rev.* **15**, 120–122 (2014). 10.1017/S1466252314000115 25358813

[32] Fulton, R. W. Bovine respiratory disease research (1983–2009). *Anim. Heal Res. Rev.* **10**, 131–139 (2009). 10.1017/S146625230999017X 20003649

[33] Theurer, M. E., Larson, R. L. & White, B. J. Systematic review and meta-analysis of the effectiveness of commercially available vaccines against bovine herpesvirus, bovine viral diarrhea virus, bovine respiratory syncytial virus, and parainfluenza type 3 virus for mitigation of bovine respiratory disease. *J. Am. Vet. Med. Assoc.* **246**, 126–142 (2015). 25517335 10.2460/javma.246.1.126

[34] Yu, J., Li, F. & Wang, D. The first decade of research advances in influenza D virus. *J. Gen. Virol.* **102**, jgv001529 (2020). 10.1099/jgv.0.001529 PMC8279278 33211641

[35] Nakatsu, S. et al. Influenza C and D viruses package eight organized ribonucleoprotein complexes. *J. Virol.* **92**, e02084–e02017 (2018). 29321324 10.1128/JVI.02084-17 PMC5827381

[36] Ishida, H. et al. Construction of an influenza D virus with an eight-segmented genome. *Viruses* **13**, 2166 (2021). 34834971 10.3390/v13112166 PMC8619389

[37] Baez, M. et al. Complete nucleotide sequence of the influenza A/PR/8/34 virus NS gene and comparison with the NS genes of the A/Udorn/72 and A/FPV/Rostock/34 strains. *Nucleic Acids Res.* **8**, 5845–5858 (1980). 7465426 10.1093/nar/8.23.5845 PMC324346

[38] Briedis, D. J. & Lamb, R. A. Influenza B virus genome: sequences and structural organization of RNA segment 8 and the mRNAs coding for the NS1 and NS2 proteins. *J. Virol.* **42**, 186–193 (1982). 6283137 10.1128/jvi.42.1.186-193.1982 PMC256059

[39] Nakada, S., Graves, P. N. & Palese, P. The influenza C virus NS gene: evidence for a spliced mRNA and a second NS gene product (NS2 protein). *Virus Res.* **4**, 263–273 (1986). 2943090 10.1016/0168-1702(86)90005-5

[40] Hongo, S. et al. Influenza C virus CM2 protein is produced from a 374-amino-acid protein (P42) by signal peptidase cleavage. *J. Virol.* **73**, 46–50 (1999). 9847305 10.1128/jvi.73.1.46-50.1999 PMC103806

[41] Pekosz, A., Lamb, R. A. & Influenza C virus CM2 integral membrane glycoprotein is produced from a polypeptide precursor by cleavage of an internal signal sequence. *Proc. Natl. Acad. Sci. U. S. A.* 95, 13233–13238 (1998). 10.1073/pnas.95.22.13233 PMC23766 9789071

[42] Hongo, S. et al. Detection of ion channel activity in xenopus laevis oocytes expressing influenza C virus CM2 protein. *Arch. Virol.* **149**, 35–50 (2004). 14689274 10.1007/s00705-003-0209-3

[43] Muraki, Y. & Hay, A. Establishment of mouse erythroleukemia cell lines expressing complete influenza C virus CM2 protein or chimeric protein consisting of CM2 and influenza A virus M2. *Acta Virol.* **53**, 125–129 (2009). 19537914 10.4149/av_2009_02_125

[44] Ishida, H. et al. Establishment of a reverse genetics system for influenza D virus. *J. Virol.* **94**, e01767–e01719 (2020). 32102883 10.1128/JVI.01767-19 PMC7199395

[45] Hutchinson, E. C., von Kirchbach, J. C., Gog, J. R. & Digard, P. Genome packaging in influenza A virus. *J. Gen. Virol.* **91**, 313–328 (2010). 19955561 10.1099/vir.0.017608-0

[46] Fujii, Y., Goto, H., Watanabe, T., Yoshida, T. & Kawaoka, Y. Selective incorporation of influenza virus RNA segments into virions. *Proc. Natl. Acad. Sci. U S A*. **100**, 2002–2007 (2003). 12574509 10.1073/pnas.0437772100 PMC149948

[47] Fujii, K. et al. Importance of both the coding and the segment-specific noncoding regions of the influenza A virus NS segment for its efficient incorporation into virions. *J. Virol.* **79**, 3766–3774 (2005). 15731270 10.1128/JVI.79.6.3766-3774.2005 PMC1075679

[48] Ozawa, M. et al. Contributions of two nuclear localization signals of influenza A virus nucleoprotein to viral replication. *J. Virol.* **81**, 30–41 (2007). 17050598 10.1128/JVI.01434-06 PMC1797272

[49] Muramoto, Y. et al. Hierarchy among viral RNA (vRNA) segments in their role in vRNA incorporation into influenza A virions. *J. Virol.* **80**, 2318–2325 (2006). 16474138 10.1128/JVI.80.5.2318-2325.2006 PMC1395381

[50] Watanabe, T., Watanabe, S., Noda, T., Fujii, Y. & Kawaoka, Y. Exploitation of nucleic acid packaging signals to generate a novel influenza virus-based vector stably expressing two foreign genes. *J. Virol.* **77**, 10575–10583 (2003). 12970442 10.1128/JVI.77.19.10575-10583.2003 PMC228515

[51] Liang, Y., Hong, Y. & Parslow, T. G. cis-Acting packaging signals in the influenza virus PB1, PB2, and PA genomic RNA segments. *J. Virol.* **79**, 10348–10355 (2005). 16051827 10.1128/JVI.79.16.10348-10355.2005 PMC1182667

[52] Ozawa, M. et al. Nucleotide sequence requirements at the 5’ end of the influenza A virus M RNA segment for efficient virus replication. *J. Virol.* **83**, 3384–3388 (2009). 19158245 10.1128/JVI.02513-08 PMC2655591

[53] Noda, T. et al. Architecture of ribonucleoprotein complexes in influenza A virus particles. *Nature* **439**, 490–492 (2006). 16437116 10.1038/nature04378

[54] Noda, T. et al. Three-dimensional analysis of ribonucleoprotein complexes in influenza A virus. *Nat. Commun.* **3**, 639 (2012). 22273677 10.1038/ncomms1647 PMC3272569

[55] Noda, T. et al. Importance of the 1 + 7 configuration of ribonucleoprotein complexes for influenza A virus genome packaging. *Nat. Commun.* **9**, 54 (2018). 29302061 10.1038/s41467-017-02517-w PMC5754346

[56] Chou, Y. et al. One influenza virus particle packages eight unique viral RNAs as shown by FISH analysis. *Proc. Natl. Acad. Sci. U S A*. **109**, 9101–9106 (2012). 22547828 10.1073/pnas.1206069109 PMC3384215

[57] Hutchinson, E. C. Influenza virus. *Trends Microbiol.* **26**, 809–810 (2018). 29909041 10.1016/j.tim.2018.05.013

[58] von Heijne, G. A new method for predicting signal sequence cleavage sites. *Nucleic Acids Res.* **14**, 4683–4690 (1986). 3714490 10.1093/nar/14.11.4683 PMC311474

[59] Ishida, H. et al. Generation of a Recombinant temperature-sensitive influenza D virus. *Sci. Rep.* **13**, 3806 (2023). 36882459 10.1038/s41598-023-30942-z PMC9992382

[60] Niwa, H., Yamamura, K. & Miyazaki, J. Efficient selection for high-expression transfectants with a novel eukaryotic vector. *Gene* **108**, 193–199 (1991). 1660837 10.1016/0378-1119(91)90434-d

[61] Killian, M. L. Hemagglutination assay for influenza virus. *Methods Mol. Biol.* **1161**, 3–9 (2014). 24899415 10.1007/978-1-4939-0758-8_1

