# Supplementary material for: Development of an influenza D virus with an eight- or nine-segment genome
Source: Sci Rep. 2026 Jan 8;16:4877. doi: 10.1038/s41598-025-34838-y (PMC12873286; doi:10.1038/s41598-025-34838-y)
Supplement: Supplementary file 1 — Supplementary Material 1 [file 41598_2025_34838_MOESM1_ESM.pdf]

Development of an influenza D virus with an eight- or nine-segment genome

Hiroho Ishida [1,*], Hironobu Murakami [1], Shuntaro Mizuno [1], Misa Katayama [2], Wataru Sekine [2], Kosuke Ohira [2], Akiko Takenaka-Uema [2], Shin Murakami [2], Makoto Nagai [1], and Taisuke Horimoto [2,*]

[1] School of Veterinary Medicine, Azabu University, Sagamihara, Kanagawa 252-5201, Japan

[2] Laboratory of Veterinary Microbiology, Graduate School of Agricultural and Life Sciences, The University of Tokyo, Bunkyo-ku, Tokyo 113-8657, Japan

*Correspondence to Hiroho Ishida, ishida@azabu-u.ac.jp

ORCID: 0000-0003-4314-199X

Supplementary Information

A **B**

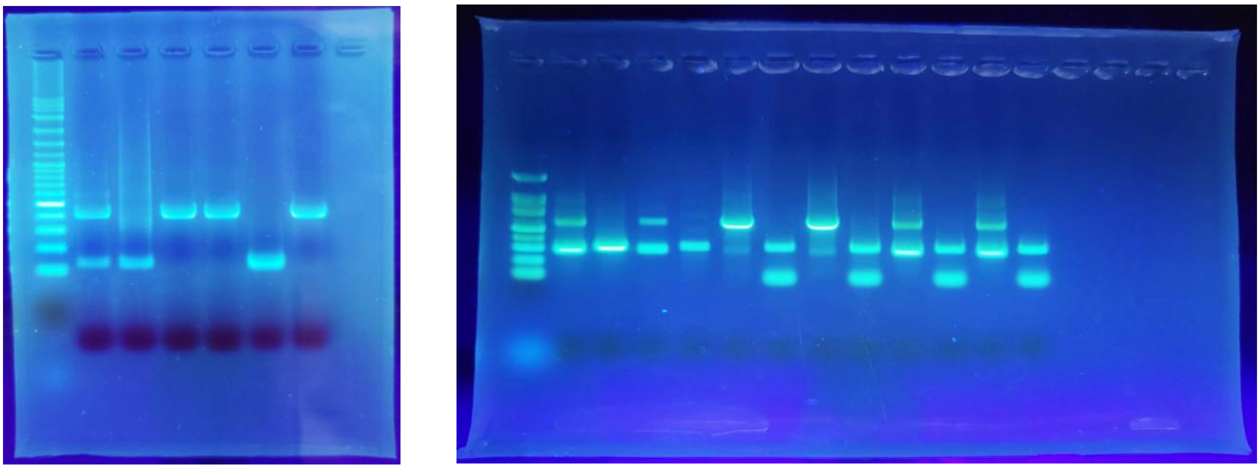

Supplementary Fig. S1 Original gel images corresponding to the main figures. (A) Gel image corresponding to Fig. 1B. (B) Gel image corresponding to Fig. 1D.

Supplementary Information

A ICV (C/Ann Arbor/1/50)

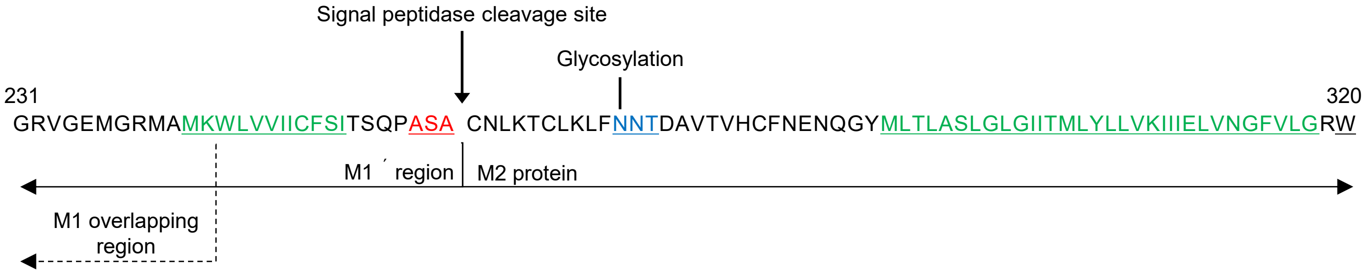

B IDV (D/swine/Oklahoma/1334/2011)

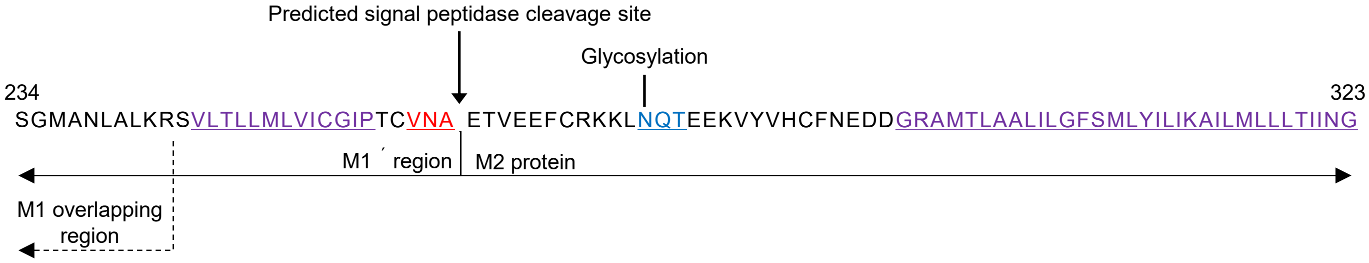

Supplementary Fig. S2 Comparison of the amino acid sequences of the P42 proteins of the influenza C virus (ICV) and influenza D virus (IDV). (A) The P42 protein of ICV is inserted into the endoplasmic reticulum via two transmembrane regions (green). On the endoplasmic reticulum side, it is cleaved into the M1′ region on the N-terminal side and the M2 protein on the C-terminal side by signal peptidase, which recognizes the signal motif sequence (red). (B) P42 protein of IDV also has two regions (purple) rich in hydrophobic amino acid regions, and these regions are believed to penetrate the endoplasmic reticulum membrane. Similarly, as observed for ICV, there is a signal motif sequence on the endoplasmic reticulum side. It is believed that P42 is cleaved by signal peptidase after this motif. The numbers indicate the positions of the amino acids at the beginning and end of the sequence. The glycosylation sequence on the endoplasmic reticulum side is presented in blue.
